# GRANDPARENTS RAISING GRANDCHILDREN IN NEW YORK STATE: UNDERSTANDING NEEDS OF A VULNERABLE POPULATION

**DOI:** 10.1093/geroni/igad104.3373

**Published:** 2023-12-21

**Authors:** Kate Guastaferro, Nusrat E Mozid, Jayna Kramsky, Matilda Melkonian, Kashyah Young, Essence Spears

**Affiliations:** New York University, New York City, New York, United States; New York University, New York City, New York, United States; New York University, New York City, New York, United States; New York University, New York City, New York, United States; New York University, New York City, New York, United States; Clark Atlanta University, Atlanta, Georgia, United States

## Abstract

Grandparents raising grandchildren are a rapidly growing population in the US – the 2020 census estimated 5.9million children < 18 were living with a grandparent and in 2023 this estimate exceeded 7.1million. The older adult was likely not anticipating raising a child and is subject to the same stressors as other adults of their age and similar socioeconomic status: financial insecurity, health problems, employment issues, and family conflict. The community-based interventions that exist for ‘grandfamilies’ address a small portion of their needs and there is no universal network of services for this family unit that can sufficiently address the grandparents’ need for support, resources, and training. A total of N = 58 grandparents (56% female; 70% White; 40% urban-dwelling; 63% married) raising grandchildren in New York State participated in the GRAND Study which sought to quantify the needs of grandparents raising grandchildren using mixed methods. Survey results, corroborated by qualitative interview and focus group data, suggest areas of need include: financial needs, legal needs (e.g., acquiring custody), social support and parenting. Additional needs were observed in grandparents’ self-reported level of depressive symptoms – 46% of responses indicated major depressive disorder – and in managing relationships with their adult child (i.e., parent of grandchild). Overwhelmingly, grandparents spoke about the ‘honor’ of raising their grandchildren despite the challenges, specifically how the public health systems with whom they interact are not responsive to their needs. Results can be used to inform a multicomponent behavioral intervention to improve the general health and well-being of grandfamilies.

